# Markers for human haematopoietic stem cells: The disconnect between an identification marker and its function

**DOI:** 10.3389/fphys.2022.1009160

**Published:** 2022-09-30

**Authors:** Beatrice Rix, Andres Hernandez Maduro, Katherine S. Bridge, William Grey

**Affiliations:** York Biomedical Research Institute, Department of Biology, University of York, York, United Kingdom

**Keywords:** haematopoietic stem cell, haematopoiesis, stem cell isolation, stem cell purification, stem cell functional markers

## Abstract

The haematopoietic system is a classical stem cell hierarchy that maintains all the blood cells in the body. Haematopoietic stem cells (HSCs) are rare, highly potent cells that reside at the apex of this hierarchy and are historically some of the most well studied stem cells in humans and laboratory models, with haematopoiesis being the original system to define functional cell types by cell surface markers. Whilst it is possible to isolate HSCs to near purity, we know very little about the functional activity of markers to purify HSCs. This review will focus on the historical efforts to purify HSCs in humans based on cell surface markers, their putative functions and recent advances in finding functional markers on HSCs.

## Introduction

The human body produces an estimated four to five billion new blood cells every day for numerous life-critical purposes, including immunity, oxygen transport and clotting at sites of bleeding (Kaushansky, 2006). The regulation of blood production must be responsive and selective, adjusting to changes in altitude (oxygen availability), hypovolemia (bleeding) and immune challenges rapidly (Zhao and Baltimore, 2015). The system of stem cells and their progeny responsible for the maintenance of blood is known as the haematopoietic system, with rare and highly potent haematopoietic stem cells (HSCs) residing at the apex and responsible for life-long blood production (Laurenti and Göttgens, 2018, Figure 1).

**FIGURE 1:**
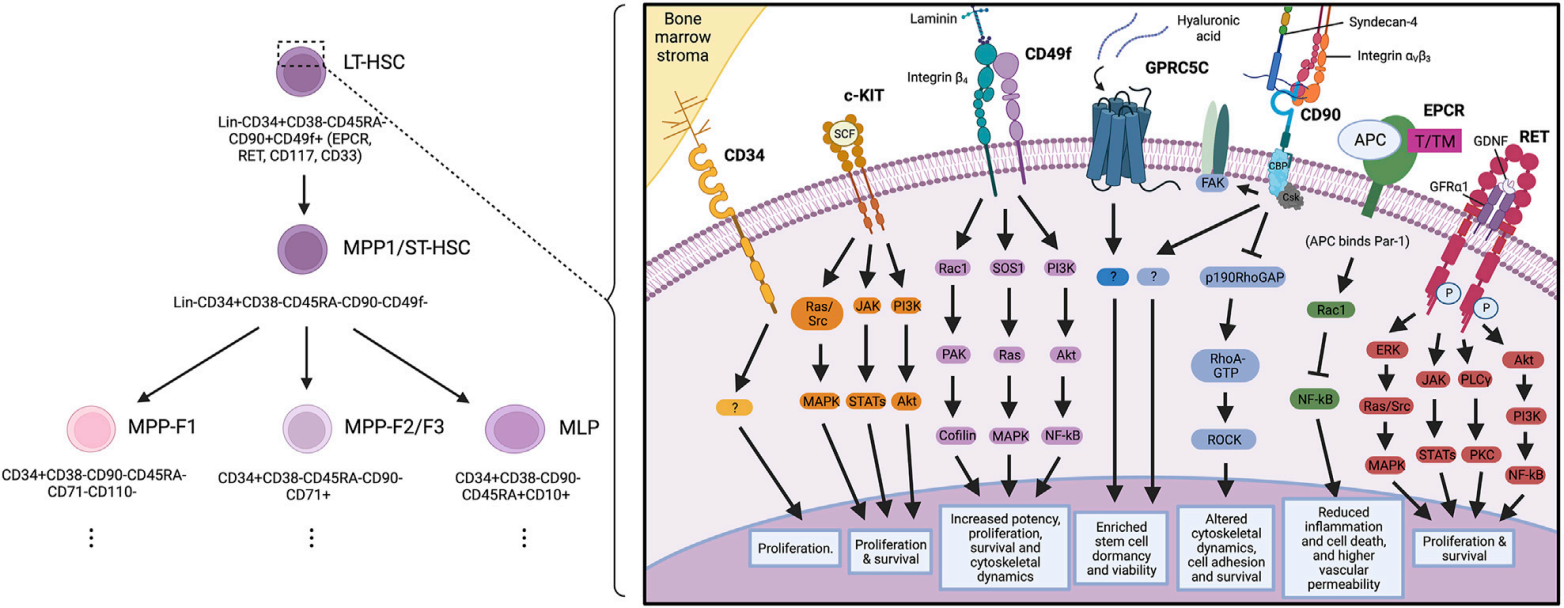
Key haematopoietic stem cell markers and their proposed signalling interactions governing haematopoietic stem cell biology.

Historically, haematopoiesis has been one of the most studied stem cell systems, leading the way in identification of hierarchical organisation, fate mapping and single cell approaches (Laurenti and Göttgens, 2018). Indeed, HSCs are defined by their ability to reform the entire haematopoietic system upon transplantation, with bone marrow transplantation (BMT) being the first regenerative approach to enter the clinic. BMT was initially used to treat patients who had received high dose radiation and suffered bone marrow failure, and as a clinical approach has since developed both regenerative and immunotherapeutic aims (Chabannon et al., 2018).

Whilst many landmark studies have revealed the origins of HSCs and the factors governing their functionality (Bertrand et al., 2010; Hamey et al., 2017; Baron et al., 2018; Hou et al., 2020), the rationale for selecting HSCs for study is based on a series of cell surface markers for which we have little annotated function. Indeed, there is still some ambiguity in the field as to which the most relevant markers to identify HSCs in different contexts (foetal, paediatric, adult, expanded) are, with the physiological function of many of these markers in HSC biology remaining unknown. As we better understand the function of key HSC markers, we may develop new insights into their biology and leverage this information to improve HSC functionality, expansion of HSCs from different sources and regenerative medicine approaches. This review focuses on the markers most widely used to identify and purify HSCs from human tissues and the current knowledge of their function (Table 1), revealed by studies in both human HSCs and cell populations from other tissues.

**TABLE 1:** Key haematopoietic stem cell markers, protein type and functional annotation.

Cell surface molecule	Gene name	Original HSC functional references	Type of protein	Functional annotation (GO terms)	
				Molecular function	Biological process
Positive selection markers (flow cytometry)					
CD34	CD34 Antigen	Civin et al., 1984. Antigenic analysis of hematopoiesis. III. A hematopoietic progenitor cell surface antigen defined by a monoclonal antibody raised against KG-1a cells	Single-pass transmembrane sialomucin protein	Protein binding. Carbohydrate binding. Sulfate binding. RNA polymerase II-specific DNA-binding transcription factor binding	Tissue homeostasis. Endothelial cell proliferation. Glomerular filtration. Endothelium development
		Katz et al., 1985. Identification of a membrane glycoprotein associated with haemopoietic progenitor cells			
		Andrews et al., 1986. Monoclonal antibody 12–8 recognizes a 115-kd molecule on both unipotent and multipotent hematopoietic colony-forming cells and their precursors			
CD90	Thy-1 cell surface antigen	Majeti et al., 2007, Identification of a Hierarchy of Multipotent Hematopoietic Progenitors in Human Cord Blood	GPI-anchored cell surface glycoprotein with a V-like Ig-domain	GTPase activator activity. Integrin binding. Protein binding. Protein kinase binding. Enzyme Building	Angiogenesis. Regulation of cell-matrix adhesion. Positive regulation of cellular extravasation. Negative regulation of protein kinase activity. Cytoskeleton organisation
		Mayani and Lansdorp, (1994). Thy-1 expression is linked to functional properties of primitive hematopoietic progenitor cells from human umbilical cord blood			
		Leyton and Hagood, (2014). Thy-1 modulates neurological cell-cell and cell-matrix interactions through multiple molecular interactions			
CD49f	Integrin subunit alpha 6	Notta et al., 2011. Isolation of Single Human Hematopoietic Stem Cells Capable of Long-Term Multilineage Engraftment	Integrin	Integrin binding. Protein binding. Insulin-like growth factor I binding. Neuregulin binding. Cadherin binding	Cell-substrate junction assembly. Cell-matrix adhesion. Integrin-mediated signalling pathway. Ectodermal cell differentiation. Cell adhesion
EPCR	Endothelial protein C receptor	Anjos-Afonso et al., 2022. Single cell analyses identify a highly regenerative and homogenous human CD34^+^ hematopoietic stem cell population	Transmembrane receptor	Protein binding. Signalling receptor activity	Blood coagulation. Negative regulation of coagulation. Hemostasis
Other markers					
RET	RET proto-oncogene	Grey et al., 2020. Activation of the receptor tyrosine kinase RET improves long-term hematopoietic stem cell outgrowth and potency	Single-pass transmembrane receptor tyrosine kinase	Protein tyrosine kinase activity. Transmembrane receptor protein tyrosine kinase activity. Nucleotide binding. Protein kinase activity. Protein serine/threonine/tyrosine kinase activity	MAPK cascade. Ureteric bud development. Neural crest cell migration. Embryonic epithelial tube formation. Protein phosphorylation
GPRC5C	G-protein coupled receptor family group 5 member C	Zhang et al., 2022. Hyaluronic acid–GPRC5C signalling promotes dormancy in haematopoietic stem cells	GPCR protein	G protein-coupled receptor activity. Protein kinase activator activity. Protein binding	G protein-coupled receptor signaling pathway. Activation of protein kinase activity. Signal transduction
CD117	KIT Proto-Oncogene (C-KIT)	Yarden et al., 1987. Human proto-oncogene c-kit: a new cell surface receptor tyrosine kinase for an unidentified ligand	Receptor Tyrosine Kinase	Nucleotide binding. Protease binding. Protein kinase activity. Protein kinase activity. Protein seine/threonine/tyrosine kinase activity. Protein tyrosine kinase activity	Ovarian follicle development. Haematopoietic progenitor cell differentiation. Myeloid progenitor cell differentiation. Lymphoid progenitor cell differentiation. Immature B cell differentiation
		Linnekin, (1999). Early signaling pathways activated by c-Kit in hematopoietic cells			
		Zhang et al., 2017. A novel slug-containing negative-feedback loop regulates SCF/c-Kit-mediated hematopoietic stem cell self-renewal			
CD133	Prominin-Like Protein 1	Cimato et al., 2019. CD133 Expression in Circulating Hematopoietic Progenitor Cells	Pentaspan Transmembrane glycoprotein	Protein binding. Cholesterol binding. Actinin binding. Cadherin binding	Retina layer formation. Photoreceptor cell maintenance. Retina morphogenesis in camera-type eye. Camera-type eye photoreceptor cell differentiation. Podocyte differentiation
	(PROM1)				
CD59	CD59 Molecule	Hill et al., 1996. High-level expression of a novel epitope of CD59 identifies a subset of CD34^+^ bone marrow cells highly enriched for pluripotent stem cells	Cell surface glycoprotein	Complement binding. Protein binding	Negative regulation of activation of membrane attack complex. Cell surface receptor signaling pathway. Blood coagulation. Regulation of complement activation. Regulation of complement-dependent cytotoxicity
CD45	Protein tyrosine phosphatase receptor type C (PTPRC)	Shivtiel et al., 2008. CD45 regulates retention, motility, and numbers of hematopoietic progenitors, and affects osteoclast remodeling of metaphyseal trabecules	Protein tyrosine phosphatase (PTP) family	Phosphoprotein phosphatase activity. Protein tyrosine phosphatase activity. Transmembrane receptor protein tyrosine phosphatase activity. Signaling receptor binding. Protein binding	MAPK cascade. Natural killer cell differentiation. Negative regulation of T cell mediated cytotoxicity. Positive regulation of T cell mediated cytotoxicity. Negative regulation of cytokine-mediated signaling pathway
GPI-80	Vanin 2 (VNN2)	Prashad et al., 2015. GPI-80 Defines Self-Renewal Ability in Hematopoietic Stem Cells during Human Development	GPI-anchored cell surface protein and pantetheinase	Protein binding. Pantetheine hydrolase activity	Nitrogen compound metabolic process. Pantothenate metabolic process
CD43	Sialophorin (SPN)	Vodyanik et al., 2006. Leukosialin (CD43) defines hematopoietic progenitors in human embryonic stem cell differentiation cultures	Transmembrane sialylated glycoprotein	Transmembrane signaling receptor activity. Protein binding. Hsp70 protein binding. Heat shock protein binding	T-helper 1 cell lineage commitment. Chemotaxis. Immune response. Cellular defense response. Negative regulation of cell adhesion
CD44	CD44 Molecule	Legras et al., 1997. CD44-mediated adhesiveness of human hematopoietic progenitors to hyaluronan is modulated by cytokines	Cell-surface glycoprotein	Transmembrane signaling receptor activity. Cytokine receptor activity. Collagen binding. Hyaluronic acid binding	Inflammatory response. Cell-matrix adhesion. Cell migration. Cytokine-mediated signaling pathway
		Cao et al., 2016. The role of CD44 in fetal and adult hematopoietic stem cell regulation			
CD9	CD9 Molecule	Fidanza et al., 2020. Single-cell analyses and machine learning define hematopoietic progenitor and HSC-like cells derived from human PSCs	Tetraspanin	Integrin binding. Protein binding	Cell adhesion. Fusion of sperm to egg plasma membrane involved in single fertilization. Negative regulation of cell population proliferation
CD48	CD48 Molecule	Sintes et al., 2008. Differential expression of CD150 (SLAM) family receptors by human hematopoietic stem and progenitor cells	SLAM-family GPI-anchored cell surface glycoprotein	Antigen binding. Protein binding. Signalling receptor activity	Regulation of adaptive immune response. Defense response. T cell activation
		Boles et al., 2011. CD48 on hematopoietic progenitors regulates stem cells and suppresses tumor formation			
CD84	CD84 Molecule	Zaiss et al., 2003. CD84 expression on human hematopoietic progenitor cells	SLAM-family cell surface glycoprotein	Protein binding. Identical protein binding	Adaptive immune response. Immune system process. Autophagy. Defense response. Cell adhesion
CD244	CD244 Molecule	Sintes et al., 2008. Differential expression of CD150 (SLAM) family receptors by human hematopoietic stem and progenitor cells	SLAM-family heterophilic receptor	Protein binding. Signaling receptor activity. MHC class I protein binding	Adaptive immune response. Natural killer cell activation involved in immune response. Immune system process. Signal transduction
		Sigurdsson et al., 2019. CD244 Marks Non-Functional Hematopoietic Stem Cells with a Mast Cell Signature after Induction of Endoplasmic Reticulum Stress			

### A unified strategy for purifying human haematopoietic stem cells?

There has already been a wide range of reports into markers present on human HSCs, with studies crossing homeostasis (Linnekin, 1999; Zaiss et al., 2003; Cimato et al., 2019), ontogeny (Vodyanik et al., 2006; Prashad et al., 2015), immune stress and disease (Boles et al., 2011; Sigurdsson et al., 2019). Whilst these studies have contributed to the HSC field, relatively few have made a long-term impact on the way we purify HSCs to study their biology. Historically, in fact, human HSC purification strategies have been through a myriad of prior iterations to find the purest population of cells to study. Each study has built upon a backbone of commonly used identification markers to purify these cells through flow cytometry. Nowadays, the most frequently used strategy amongst researchers is lineage marker negative (Lin^−^)CD34^+^CD38^−^CD45RA^−^CD90^+^CD49f^+^ (Notta et al., 2011). Recent research efforts from the groups of Prof. Bonnet and Dr. Cabezas-Wallscheid have begun to challenge this strategy with the addition of EPCR *in lieu* of CD90 (Anjos-Afonso et al., 2022) and potentially GPRC5C to mark dormant HSCs from active HSCs (Y. W. Zhang et al., 2022), leading to a new strategy of Lin^−^CD34^+^CD38^−^CD45RA^−^CD49f^+^EPCR^+^ that displays a stem cell frequency of approximately 1 in 5 cells. It is from this strategy that we review key markers and their putative biological functions in HSCs.

### CD34

The single-pass transmembrane sialomucin protein CD34 is expressed on HSCs and progenitors throughout ontogeny, from the early stages of foetal development to adult bone marrow (Civin et al., 1984; Katz et al., 1985; Sidney et al., 2014), embryonic fibroblasts (Brown et al., 1991) and endothelial cells (Fina et al., 1990). Although a very rare CD34 negative population of HSCs is known to be hierarchically above CD34 positive cells in umbilical cord blood, adult haematopoiesis is sustained by CD34 positive haematopoietic stem and progenitors cells (Anjos-Afonso et al., 2013). When haematopoietic stem cell transplantation is used as a regenerative medicine approach, CD34 is the critical marker monitored for stem cell dose in donor cells before transplantation. Indeed, it is the key criteria used to select umbilical cord blood donors with sufficient HSCs for transplantation and is one of the best predictors of long-term reconstitution (Chabannon et al., 2018).

Little is known about the function of CD34 on human HSCs, though many putative functions have been suggested in haematopoietic cells. Ectopic expression of human CD34 in murine haematopoietic cells indicated a role in adherence to bone marrow stroma (Baumhueter et al., 1993; Healy et al., 1995), but later studies of haematopoiesis in avian models suggested the opposite (reviewed elsewhere: Nielsen et al., 2002). This is likely due to the altered glycosylation patterns on cell types used in these studies. Early studies argued for CD34 to be an inhibitor of HSC proliferation (Fackler et al., 1995; Sassetti et al., 2000), yet later studies of CD34^−^ HSCs demonstrated that umbilical cord blood derived CD34^+^ HSCs have higher proliferative potential in primary transplant recipients than CD34^−^ HSCs (Anjos-Afonso et al., 2013). This may be in part due to upregulation of CD34 cell surface expression after phosphorylation by protein kinase C (Fackler et al., 1990, 1992). There has been little investigation into the function of CD34 in recent years, and with our current knowledge of its function, this leaves it as a molecule solely for identification of functional cell subsets, with no clear functional role in HSCs.

### CD90 (Thy-1)

CD90 is a glycophosphatidylinositol (GPI) anchored cell surface protein with a V-like Ig-domain. It was demonstrated to enrich for functional HSCs through mouse *in vivo* studies in 2007 and has since been a commonly used marker to identify human HSCs (Majeti et al., 2007). CD90 is also expressed in endothelial cells and smooth muscle cells (Bradley et al., 2009). Although there have been no reports of CD90 functionality in human HSCs, much work has been done to understand CD90 function in other systems.

CD90 may signal in *cis* or *trans* (depending on the context) with SRC family kinases, G inhibitory proteins, GTPases and tubulin (Rege & Hagood, 2006; Avalos et al., 2009; Wandel et al., 2012). The extracellular domain of CD90 has been shown to interact with integrins αv/β3 (Kong et al., 2013) and αx/β2 (Leyton and Hagood, 2014), as well as Syndecan-4 (Kong et al., 2013). These interactions can modulate PKCα, FAK, RhoA/ROCK and cell survival pathways. Although the majority of these findings have been in non-haematopoietic cell types, the pathways implicated have all been previously shown to be important for HSC functionality. Early reports in HSCs argued for CD90 maintaining quiescence in stem cells to improve functionality (Mayani and Lansdorp, 1994; Majeti et al., 2007). Interestingly, this may be critical for CD90-dependent maintenance of cancer stem cells in other systems and the role of CD90 in cancer has been well reviewed elsewhere (Sauzay et al., 2019). With recent research efforts potentially removing CD90 from HSC purification strategies, it will be interesting to see if CD90 is required for maintaining HSCs long-term during regenerative medicine approaches and which HSC functions are gained or lost in the absence of CD90.

### Integrin subunit alpha 6 (CD49f)

The integrin alpha chain subunit protein, CD49f, was identified in 2011 as a marker that could be used to further purify the Lin^−^CD34^+^CD38^−^CD45RA^−^CD90^+^ HSC population to 1 in 10.5 HSC frequency (Notta et al., 2011). CD49f has been identified as a biomarker of stem cells across systems, including epithelial tissues, cardiac, embryonic and neuronal stem cells. This has been well reviewed elsewhere (Krebsbach and Villa-Diaz, 2017). Integrins are type I transmembrane glycoproteins with 18 subunits in the alpha family and 8 in the beta family, leading to potentially 24 different heterodimer combinations that can bind extracellular matrix components (Hynes, 2002). In this way, integrins can govern signalling mechanisms regulating differentiation, gene expression, motility, polarity and proliferation (Watt, 2002).

In both cancer and development, CD49f can act as a receptor for laminins, with loss of either laminins or deletion of *CD49f* leading to alterations in breast cancer cell survival (Goel et al., 2014), cerebral malformations and skin blistering in mice (Georges-Labouesse et al., 1996). Albeit nothing is known of the function of CD49f in HSCs. The potential for binding extracellular matrix components and niche engagement is intriguing. *CD49f* is regulated by hypoxia inducible factors, which are highly upregulated in the HSC niche (Brooks et al., 2016), potentially offering a functional response to correct niche localisation. Further regulators of *CD49f* expression include positive regulation by Oct4 and Sox2 in mesenchymal stem/stromal cells (Yu et al., 2012) and negative regulation by KLF9 in glioblastoma stem cells, which is important for repressing *CD49f* expression and glioblastoma stemness (Ying et al., 2014). These studies identified FAK as a potential downstream target of CD49f, but this has not been investigated in more detail in HSCs.

### Endothelial protein C receptor (EPCR/CD201)

The type I transmembrane protein Endothelial Protein C Receptor (EPCR) is a transmembrane glycoprotein capable of binding to protein C and activated protein C (Fukudome and Esmon, 1994). EPCR was first reported to be expressed on blood vessel endothelium, liver endothelium and splenic cells (Laszik et al., 1997). It was later found to be expressed in a wide variety of cell types, including monocytes, neutrophils, keratinocytes, cardiomyocytes and neurons (Gleeson et al., 2012). EPCR was subsequently described to mark HSCs that had expanded during *in vitro* culture studies (Fares et al., 2017). More recently, Anjos-Afonso and others reported EPCR-positive HSCs to sit at the top of haematopoietic hierarchy, able to give rise to all the immunophenotypic HSC populations previously reported upon transplantation (Anjos-Afonso et al., 2022). These findings have been particularly exciting as it is one of the first reports of a purifying cell surface marker conserved across human and mouse HSCs. Whilst there has been no investigation into the function of EPCR on human HSCs, Anjos-Afonso and others report EPCR-positive HSCs to be slow cycling, have low metabolic features and enriched for multi-lineage transcriptional programmes such as *HOXA6*, *BCL6* and *CEBPA* expression (Anjos-Afonso et al., 2022).

The function of EPCR has been well researched in other cell types, with the binding of EPCR to protein C known to support thrombin-TM protein C activation and facilitating blood coagulation (Stearns-Kurosawa et al., 1996). EPCR may also facilitate the cleavage of other cell surface/secreted factors, including protease activated receptor 1 (PAR1) and PAR2 and human factor X (Schuepbach and Riewald, 2010; Disse et al., 2011), again contributing to coagulation. This could indicate a link between HSC surveillance of coagulation and a potential rapid response modulated by EPCR.

Soluble EPCR has been shown to bind to activated neutrophils and monocytes (Kurosawa et al., 2000; Fink et al., 2013), in turn leading to the cleavage of EPCR and its subsequent degradation. There has been significant interest in recent years surrounding inflammatory myeloid cells and clonal haematopoietic populations (Cull et al., 2017). The majority of work has identified second messengers for modulating this dynamic. As such, if direct interactions occur *via* EPCR, there could still be much to learn about the regulation of HSCs by pre-leukaemic immune cells.

EPCR may also play a role in intracellular signalling in HSCs. Reports of EPCR regulating the RhoA/Rac1/NFκB axis in concert with PAR1 and in response to APC could result in anti-inflammatory and anti-apoptotic signalling cascades that are present in HSC transcriptomes (Joyce et al., 2001; Riewald et al., 2002; Mosnier et al., 2007). Together, these studies offer valuable insights to the potential function of EPCR in human HSCs, and (with validation directly in HSCs) may offer new targets for improving state-of-the-art expansion of human HSCs.

### Rearranged during transfection (RET)

The receptor tyrosine kinase, RET, is a single pass transmembrane protein that is activated by glial family ligands (GLFs) such as the glial derived neurotrophic factor (GNDF) and its co-receptor GFRα1 (Mulligan, 2014). We previously reported RET expression on human HSCs from umbilical cord blood, with significant enrichment on the CD49f+ HSCs (Grey et al., 2020). RET has been well documented to govern a multitude of downstream signalling pathways, including JAK/STAT, ERK/AKT, RAC1 and PI3K, amongst many others (Mulligan, 2014).

We reported that the functional activity of RET is enriched in the stem and early progenitor population of umbilical cord blood-derived haematopoietic cells; and that, when activated during *in vitro* expansion protocols, RET drives a wide variety of key signalling pathways (Figure 1). This results in improved proliferation, survival and increased oxidative stress response in HSCs, ultimately culminating in improved expansion of HSCs for transplantation (Grey et al., 2020).

Recent work focusing on the bone marrow microenvironment has demonstrated that cholinergic signals preserve HSCs during regenerative stress in mice (Fielding et al., 2022). Similar to CD49f+ HSCs residing in hypoxic niches that drive *CD49f* expression, RET^+^ HSCs may be enriched in specialised niches that support HSCs through GFLs.

### G protein-coupled receptor class C group 5 member C (GPRC5C)

Recent work from the Cabezas-Walscheid group has identified the orphan receptor G Protein-Coupled Receptor Class C Group 5 Member C (GPRC5C) as a marker of dormant human HSCs (Y. W. Zhang et al., 2022). Through elegant *in vivo* studies, the team demonstrated that GPRC5C is essential for long-term HSC function and to maintain quiescence and stemness.

GPRC5C is a seven-pass transmembrane protein with little known function (Rajkumar et al., 2018). Indeed, reports in mouse liver demonstrate that Gprc5c is pH sensitive and may regulate Na+/H+ exchanger 3 (NHE3) activity (Rajkumar et al., 2018). Zhang and others screened GPRC5C for potential binders and demonstrated that hyaluronic acid (HA) is a ligand of GPRC5C (Zhang et al., 2022). Treatment of HSCs with GPRC5C resulted in a maintenance of quiescence, including the expression of dormancy associated genes (*Gprc5c*, *Rarb* and *Cyp26b1*).

The signalling intermediates that modulate the response of HSCs to HA through GPRC5C have yet to be elucidated, and whether HA treatment is compatible with *in vitro* HSC expansion protocols has yet to be tested. Future research into the mechanism of GPRC5C regulation of human HSCs has the potential to shed light on key factors required to maintain HSCs long-term.

## Discussion

The ever-evolving field of HSC purification has led the way in our understanding of stem cell biology and developing single cell approaches to study stem cells. Historically, efforts have been directed towards purifying cells with increasing stem cell potential and studying their biology (e.g. CD34, CD90, CD49f). More recently, a new wave of function-led discovery through transcriptomic (GPRC5C) and proteomic (RET) efforts have begun to reveal the function of identification markers. This is a critically important step forward, as the potential for an identification marker to have critical functions for HSCs provides new therapeutic targets that could be leveraged in regenerative medicine approaches.

Here we have reviewed the key cell surface markers used to identify HSCs in humans. Other biomarkers exist but are rarely used to purify HSCs for functional assessment. These include CD33, which was identified in an elegant single cell transplantation study by the Eaves lab (Knapp et al., 2018) and historical studies from the Bonnet lab (Taussig et al., 2005). Taussig et al. raise the issue of conserved markers between healthy HSCs and malignant haematopoietic cells (in this case acute myeloid leukaemia cells). Indeed, without robust markers of HSCs throughout ontogeny and disease it is difficult to intervene to protect HSCs during bone marrow failure or treatment related depletion. When considering the effect of niche derived signals (both in a healthy or leukaemic scenario), it is vitally important to know which markers of HSCs may respond to niche-derived signals to better understand how the niche shapes HSC functionality in health and disease (Batsivari et al., 2021). We have recently reported a new Frontier in proteostatic targeting to selectively deplete leukaemic stem cells whilst preserving healthy HSCs (Grey et al., 2018; Grey et al., 2022), but this has relied on intracellular mechanisms and does not aid purification of healthy HSCs from leukaemic patients. In particular, the expression of CD117 (cKIT), Wnt responsive receptors or CD123 would suffer from the same inability to distinguish healthy HSCs from leukaemic cells.

With state-of-the-art HSC culture protocols developing at a rapid rate (Fares et al., 2014; Grey et al., 2020; Y. W. Zhang et al., 2022) and a new methodology from mouse HSCs on the horizon for human HSCs (Wilkinson et al., 2019), it is critical that we understand what the most relevant cells to expand are for regenerative medicine approaches. Additionally, donor cells used for expansion protocols may have a different absolute number of CD34^+^ cells, but this will not necessarily always correlate with their total HSC content or ability to reconstitute conditioned recipients long-term. In light of this, higher homogeneities in HSC populations for study—as well as a better understanding of the function of purification markers—will provide critical information in the field of HSC biology and further leverage current regenerative medicine approaches.

BOX 1 **State of the art: expansion of human HSCs**

The accessibility of umbilical cord blood (UCB) as a source of human HSCs has fuelled the development of *ex-vivo* expansion techniques to increase the yield of these rare cell types for further downstream therapeutic applications. Methods for expanding CD34^+^ cells from UCB, including treatment with UM171, a small molecule HSC self-renewal agonist, have shown a median 49-fold expansion of CD34^+^ cells and promising clinical outcomes (Cohen et al., 2020; Dumont-Lagacé et al., 2022). The limitations of these methods, however, are the inherent variability in quality of HSCs expanded in culture. Recent studies have identified that this variability can be significantly mitigated by removing serum (namely bovine serum albumin) from expansion culture conditions (Ieyasu et al., 2017). The current gold-standard for *ex-vivo* culture of human HSCs (Lin^−^CD34^+^CD38^−^CD45RA^−^CD90^+^CD49f^+^) from UCB affords on average 7 days of *ex-vivo* culture for experimental assays, with a 3-fold expansion of this population that peaks at around 4 days (Keyvani Chahi et al., 2022). Sudo et al. recently applied their polyvinyl alcohol (PVA)-based culture conditions, which facilitate up to 800-fold expansion of long-term mouse HSCs (Wilkinson et al., 2019) to human HSCs and display a modest improvement of yield over current human HSC *ex-vivo* culture conditions of up to 4.5-fold expansion over 14 days (Sudo et al., 2021). Whilst this study did not carry out as robust *in vivo* assessment as current state of the art approaches (Fares et al., 2014), it does provide optimism for translating this technology to the human system. Currently, it is proposed that PVA stabilises cytokines present in culture media, and that it may provide additional mechanical or chemical stimuli to support LT-HSCs. However, the identification of the function of human HSC markers will aid in the demystification of how to consistently and robustly expand human HSCs for allogeneic transplantation.
